# The potential of developing high hepatic clearance drugs via controlled release: Lessons from Kirchhoff’s Laws

**DOI:** 10.1016/j.jconrel.2024.07.040

**Published:** 2024-07-29

**Authors:** Leslie Z. Benet, Markus Ville Tiitto, Jasleen K. Sodhi

**Affiliations:** Department of Bioengineering and Therapeutic Sciences, Schools of Pharmacy and Medicine, University of California San Francisco, San Francisco, CA, USA

**Keywords:** Clearance, Absorption, Kirchhoff’s Laws, Pharmacokinetics, Controlled release

## Abstract

When a new molecular entity is predicted to exhibit high clearance in humans, pharmaceutical sponsors almost universally search for similar acting back-up compounds that will demonstrate low clearance. Here we show that, except for oral dosing, there can be marked advantages to developing and commercializing controlled release formulations of high clearance drugs, the expertise of readers of this journal. Our recent publications demonstrate that the universally held pharmacokinetic principle that drug delivery rate has no effect on measured drug clearance is not correct. Rather, we show that if clearance from the drug delivery site is markedly less than the iv bolus clearance of a drug, the in vivo drug clearance can be the drug delivery clearance controlled by the designed dosage form. This approach will be especially advantageous for high hepatic clearance drugs. These advantages include not being concerned with: a) saturable nonlinear kinetics, b) significant pharmacogenomic differences, c) drug-drug induction mechanisms, and d) in many cases drug-drug inhibition interactions. This is due to the ability of a drug sponsor to design clearance, independent of the pharmacokinetic characteristics for high clearance compounds, where clearance from the dosage form becomes the drug clearance from the patient. Recognition of this principle, as described here, results from our development of the use of Kirchhoff’s Laws from physics to derive rate-defining clearance and rate constant elimination processes independent of differential equation derivations. The key message for readers of this journal is that high clearance drugs are potentially drugable through formulation design and should not be outright disregarded, since for such drugs the dose-corrected area under the curve can be increased if the release rate from the injection site is controlled and slow resulting in drug clearance from the body controlled by clearance from the dosage form. The concepts presented here describe previously unrecognized advantages of controlled release formulations.

## 1. Introduction

It is generally believed that high clearance drugs, drugs where the clearance approaches the blood flow to the organ of elimination, cannot be developed as successful therapeutic agents. For such molecules, even those exhibiting a marked positive therapeutic pharmacodynamic response and negligible toxicity, sponsors seek backup molecules exhibiting markedly lower clearance. There is no doubt that it is not feasible nor reasonable to pursue development of an orally dosed high hepatic clearance drug. However, often this truth is implemented to suggest that when a potentially effective drug is found to be high hepatic clearance, further development should be discontinued. Here we show, based on Kirchhoff’s Laws, that there are significant advantages to pursuing the development of potentially effective high hepatic clearance drugs. The overall message of this publication is that the universally held belief that measured drug clearance is independent of input pharmacokinetics is not true. Thus, drug delivery scientists can formulate a nonoral dosage form where the clearance from the delivery site will be the drug clearance observed in vivo, thereby controlling the in vivo drug pharmacokinetics.

## 2. The history

Pharmacokinetics was established based on differential equations for first-order processes for concentration measurements, as an extension of approaches in chemistry that define rates of reaction for amount measurements. It is generally believed, since the initiation of pharmacokinetics, that dosing a drug via an alternate route (AR), as opposed to an iv bolus dose, leads to Eq. (1)

(1) $$AUC_{0\to \infty ,AR}=\frac{F•\text{Dose}}{CL_{AR}}$$

where $AUC_{0\to \infty ,AR}$ is the systemic exposure as measured by the area under the concentration-time curve over all time following dosing via the AR, F is the bioavailability of the drug via the alternate route, and $CL_{AR}$ is the clearance of drug from the systemic circulation following dosing via the AR. When we administer an iv bolus dose, the equivalent of Eq. (1) becomes

(2) $$AUC_{0\to \infty ,\text{iv bolus}}=\frac{\text{Dose}}{CL_{\text{iv bolus}}}$$

Every pharmacokinetic textbook and all teaching of pharmacokinetics today assume that $CL_{AR}=CL_{\text{iv bolus}}$. The implication is that input processes have no effect on AUC and that F cannot exceed 1.0. That is, no matter how slow the input process (the rate of absorption from an alternate site or the rate of infusion), the clearance measure in Eq. (1) will equal the clearance measure in Eq. (2).

In a recent publication [1], we provide examples of statistically significant instances where bioavailability is >1 for drugs with linear kinetics, where statistically significant lower renal clearances were observed following alternate routes of administration compared to iv bolus drug dosing, and where bioavailability calculated by excretion of unchanged drug in the urine was statistically significantly smaller than bioavailability calculated using systemic concentrations in the same study. None of these outcomes are possible if $CL_{AR}=CL_{\text{iv bolus}}$ is invariably true, since such measured outcomes result from a very slow input process increasing the measured systemic AUC.

We pointed out [2,3] that the assumption of $CL_{AR}=CL_{\text{iv bolus}}$ results from the use of differential equations, as is done in chemistry, to derive the amount of drug in the systemic circulation as a function of rate constants to determine the relationship between input and elimination. These derived amount equations are divided by a volume of distribution to convert amounts into concentrations, and integration over all time then yields AUC values (Eqs. (1 and 2)). However, only one volume term can be input into an amount differential equation, which is justifiable in chemistry where all reactions occur in a fixed fluid volume, but is not valid in pharmacokinetics, pharmacology and clinical medicine where the volume of distribution at the input site will not be the systemic circulation volume of distribution, or where the volumes of distribution of the drug and its subsequent metabolites will not be identical in vivo. Having re-evaluated the derivation of drug clearance via the use of Kirchhoff’s Laws to account for these limitations, we propose that the applications of high clearance drug candidates may also be expanded beyond their currently accepted potential in clinical drug development.

## 3. Application of Kirchhoff’s Laws to derive clearance for in series processes

In 2022, we discovered that applications of Kirchhoff’s Laws from physics would provide a pathway to derive clearance and overall rate constants for processes in series independent of differential equation derivations [3]. We showed that consistent with Kirchhoff’s Laws for processes in series, the inverse of the overall clearance or the overall rate constant would equal the sum of the inverse of the individual rate defining processes entering and the inverse of the individual rate defining process leaving. Here for clearance

(3) $$\frac{1}{CL_{\text{overall}}}=\frac{1}{CL_{\text{entering rate}-\text{defining process}}}+\frac{1}{CL_{\text{leaving rate}–\text{defing process}}}$$

We used this relationship to derive hepatic clearance [3] where the entering clearance is hepatic blood flow and the leaving clearance is the intrinsic clearance multiplied by the fraction of drug unbound in the blood. However, for this Perspective the relevant rate-defining processes are clearance from the input site (entering clearance) and total body clearance following an iv bolus dose (leaving clearance). Therefore, since $CL_{\text{overall}}$ is the measured clearance from the alternate route, $CL_{AR}$

(4) $$\frac{1}{CL_{AR}}=\frac{1}{C{\text{L}}_{\text{from the input site}}}+\frac{1}{CL_{\text{iv bolus dose}}}$$

Solving Eq. (4) gives

(5) $$CL_{AR}=\frac{CL_{\text{iv bolus dose}}}{1+\frac{CL_{\text{iv bolus dose}}}{CL_{\text{from the input site}}}}$$

Examination of Eq. (5) demonstrates that if $CL_{\text{from the input site}}\gg CL_{\text{iv bolus dose}}$, the second term in the denominator approaches zero and then $CL_{AR}=CL_{\text{iv bolus}}$. And this is frequently the case, especially for orally dosed drugs where often the absorption clearance from the gut is much greater than the iv bolus clearance of the drug.

However, if $CL_{\text{from the input site}}\ll CL_{\text{iv bolus dose}}$, then $CL_{AR}=CL_{\text{from the input site}}$. Administration delivery rate calculations have traditionally been guided by the differential equation-determined systemic clearance, $CL_{\text{iv bolus dose}}$, believing this would determine peak/trough and average steady-state concentrations of the drug candidate within the range of the therapeutic index for safety and efficacy. Of particular relevance to the field of drug delivery, the Kirchhoff’s Laws derivation indicates that drug delivery rates will be determined by the slowest clearance parameter, which can be $CL_{\text{from the input site}}$. Specifically, what we propose based on the Kirchhoff’s Laws derivation (Eq. (5)) is that the goal of the design of a successful drug delivery system should be that clearance from the body will be only a function of clearance from the delivery site, i.e., $CL_{\text{from the input site}}\ll CL_{\text{iv bolus dose}}$. Then the proper drug delivery rate for such a delivery device is the product of the desired steady-state concentration (C_ss_) multiplied by the clearance of drug from the delivery device. Thus, this approach is very amenable to high clearance drugs.

## 4. The case for the development of high hepatic clearance drugs exhibiting promising pharmacodynamics

### 4.1. The positives

There are a number of positive characteristics of high hepatic clearance drugs that have not received attention. First, the clearance of high hepatic clearance drugs will predominantly be affected by hepatic blood flow, thus these drugs are not expected to exhibit clinically relevant nonlinear elimination. (See the Appendix for a mathematical exercise illustrating this point.) Secondly, for the same reason, pharmacogenomic differences from patient to patient, and even the relevance of the metabolizing enzyme will not be clinically important. Therefore, metabolic drug-drug inhibition interactions (DDIs) will not be a concern unless the interaction clearance following an iv bolus dose (${CL}_{\text{iv bolus dose}-DDI}$) begins to approach the slow clearance from the input site as shown in Eq. (7), which is derived from Eqs. (4 and 5) with the systemic DDI-affected clearance ${CL}_{\text{iv bolus dose}-DDI}$ substituted for $CL_{\text{iv bolus}}$.


(7) $$CL_{AR-DDI}=\frac{CL_{\text{iv bolus dose}-DDI}}{1+\frac{CL_{\text{iv bolus dose}-DDI}}{CL_{\text{from the input site}}}}$$

Metabolic induction DDIs will never be a problem since they could only increase the iv bolus clearance for a drug that is already high clearance.

We also suspect that high hepatic clearance drugs will be predominantly highly soluble and thus easier to formulate. This supposition is based on our observation that when drugs are classified using the Biopharmaceutics Drug Disposition Classification System (BDDCS) [4], the Class 1, highly soluble - extensively metabolized drugs, do not exhibit clinically-relevant transporter effects. We hypothesized that although BDDCS Class 1 drugs may be shown to be transporter substrates in vitro, in vivo the passive membrane passage of a high permeability – high solubility drug may be markedly higher than the active transport contribution. As a simple test of this hypothesis, we examined the BDDCS class of drugs [4] with published clearances >15 mL/min/kg (the ER being approximately >0.70) as reported in the 13th Goodman and Gilman edition [5]. We found that for 24 drugs with ER>0.70, 79% were BDDCS class 1 potentially supporting our hypothesis.

Fourth, high clearance drugs could be interesting in terms of targeted drug delivery. When a high clearance drug is targeted to a site of pathology and locally delivered and released for activity by a circulating drug delivery system (e.g., a nanoparticle) then all other drug that does not end up at the site and is prematurely released in the circulation (or released from secondary uptake organs) will be efficiently cleared before it becomes systemically active (and thus toxic). This could increase the therapeutic index.

Finally, fifth, and most important for readers of this journal: For high clearance drugs, it is more easily possible to design slow AR delivery systems that will define the in vivo clearance of the drug compared to low clearance drugs. When $CL_{\text{iv bolus}}\gg CL_{\text{from the input site}}$ (Eqs. (4 and 5)) the measured $CL_{AR}$ will also reflect the slower entering clearance from the input site, and that clearance can be controlled by the formulator in the development of the AR formulation. That is, high drug clearance, as measured following iv dosing, is irrelevant since the clearance will be prescribed by release from the formulation.

### 4.2. The negatives

Now there are some obvious potential problems with our proposal to increase the development of high hepatic clearance drugs. Foremost, as stated above, this will not be a useful approach when developing oral dosage forms, since high clearance drugs will exhibit very large first pass hepatic and potentially intestinal metabolism, yielding very low and probably highly variable bioavailability. Second, although AR dosing of high clearance drugs will not be affected by differences in metabolism, they will be subject to changes in hepatic blood flow, as demonstrated by the effect of concomitant propranolol dosing on the pharmacokinetics of the high clearance drug lidocaine following iv infusion dosing in humans [6] and iv bolus and iv infusion dosing in dogs [7]. Reduced hepatic blood flow in advanced liver cirrhosis can still lead to drug-disease interactions by decreasing clearance via impaired access to hepatic metabolism sites. However, significant drug-drug interactions affecting blood flow are much rarer than metabolic interactions. Third, it is possible that the variability of the entering clearance of drug from the alternate sites may be too great to be a useful therapeutic approach. Yet, variability of elimination is probably much greater than the variability of delivery from a well-designed drug release formulation. Fourth, for these AR formulations to be effective, a sufficient amount of the drug needs to be delivered within a relatively low volume formulation, requiring potent compounds to be effective. But this is true independent of drug clearance values.

## 5. Conclusions

The key message for readers of this journal is that high clearance drugs are potentially drugable through formulation design, and should not be outright disregarded since for such drugs the dose-corrected area under the curve can be increased (compared to immediate release formulations) if the release rate from the injection site is controlled and slow, whereby the clearance of drug from the patient will be the clearance from the dosage form. We believe that the incorrect assumption (based on the differential equation approach) that leads to $CL_{AR}=CL_{\text{iv bolus}}$, results in the conclusion that one cannot escape the negative consequences of high hepatic clearance and may have led to the abandonment of a number of potentially highly useful therapeutics. Here we illustrate that any nonoral slow release formulation can overcome the negative effects of high hepatic clearance. Today, there is an increased use of subcutaneous commercial formulations for highly effective drugs [8]. We believe that this and other AR formulations may prove therapeutically beneficial for high hepatic clearance drugs. In our opinion the advantages of such an approach (no concern for metabolic and pharmacogenomic characteristics of drug elimination; no concern for induction DDIs; probable decreased concern for inhibitory metabolic DDIs; most high clearance compounds are highly soluble facilitating ease of formulation; the advantage for targeted delivery that drug released elsewhere will be rapidly eliminated; and most significant the ability of the drug sponsor to control the drug clearance as a function of the designed delivery system) outweigh the potential negatives. Finally, readers will recognize that if slow clearance from the delivery site for an AR dosage formulation can increase the dose corrected AUC compared to an iv bolus dose this could also be true in comparing dose corrected AUC for extended release (ER) oral dosage forms versus immediate release (IR) oral dosage forms. Then, since for the great majority of drugs an increase in AUC can result in an increase of the pharmacodynamic effect, as we recently presented in our published abstract [9; the full poster is included as Supplementary Material; This poster may also be accessed via the Clinical Pharmacology and Therapeutics 2024 meeting web site], we can explain multiple published studies demonstrating increased pharmacodynamic response for equivalent dose ER vs IR formulations as we will describe in detail in a future publication.

## Supplementary Material

Supplementary material

## Data Availability

Data for drugs that are high clearance and BDDCS classification may be found in references 4 and 5.

## Appendix A. Appendix

In our Kirchhoff’s Laws publications [1–3,10,11] we showed that when hepatic basolateral transporters are not clinically relevant, the general hepatic blood clearance (CL_H_) equation, previously believed to be the well-stirred model, is

(A1)
$$CL_{H}=Q_{H}•ER=Q_{H}•\frac{f_{uB}•CL_{int}}{Q_{H}+f_{uB}•CL_{\text{int}}}$$

where Q_H_ is the hepatic blood flow, taken to be 90 L/h in a 70 kg human, f_uB_ is the fraction unbound in blood and CL_int_ is the intrinsic clearance. For a high clearance drug, where $f_{uB}•CL_{int}\gg Q_{H}$, Eq. (A1) simplifies to $CL_{H}=Q_{H}$, illustrating that changes in $f_{uB}•CL_{int}$ will have little impact on total hepatic clearance values. Using numerical values, consider a high clearance drug where V_max_ is 9 g/h and K_M_ is 0.004 g/L. Then since f_uB_·CL_int_ is equal to $\frac{\text{Vmax}}{K_{M}+C}$ [12], one can determine CL_H_ at a low substrate concentrations (C = 0.0005 g/L) from Eq. (A1). The value is 86.1 L/h. This is a high clearance drug with an ER = 0.957. Now consider the clearance value when C is 0.01 g/L, 2.5-fold higher than K_M·_ CL_H_ = 78.9 L/h and ER = 0.877. That is, although the systemic concentration increased by 2000% to levels significantly exceeding K_M_, CL_H_ only decreased by 8.4%, since as stated in the text “the clearance of high hepatic clearance drugs will predominantly be affected by hepatic blood flow, thus these drugs are not expected to exhibit clinically relevant nonlinear elimination” and aspects related to individual metabolic enzymes will not be relevant.
