# Supplementary material for: The potential of developing high hepatic clearance drugs via controlled release: Lessons from Kirchhoff’s Laws
Source: J Control Release. Author manuscript; Available in PMC 2025 Sep 1. (PMC11731068; doi:10.1016/j.jconrel.2024.07.040)
Supplement: Supplementary material [file NIHMS2045676-supplement-Supplementary_material.docx]

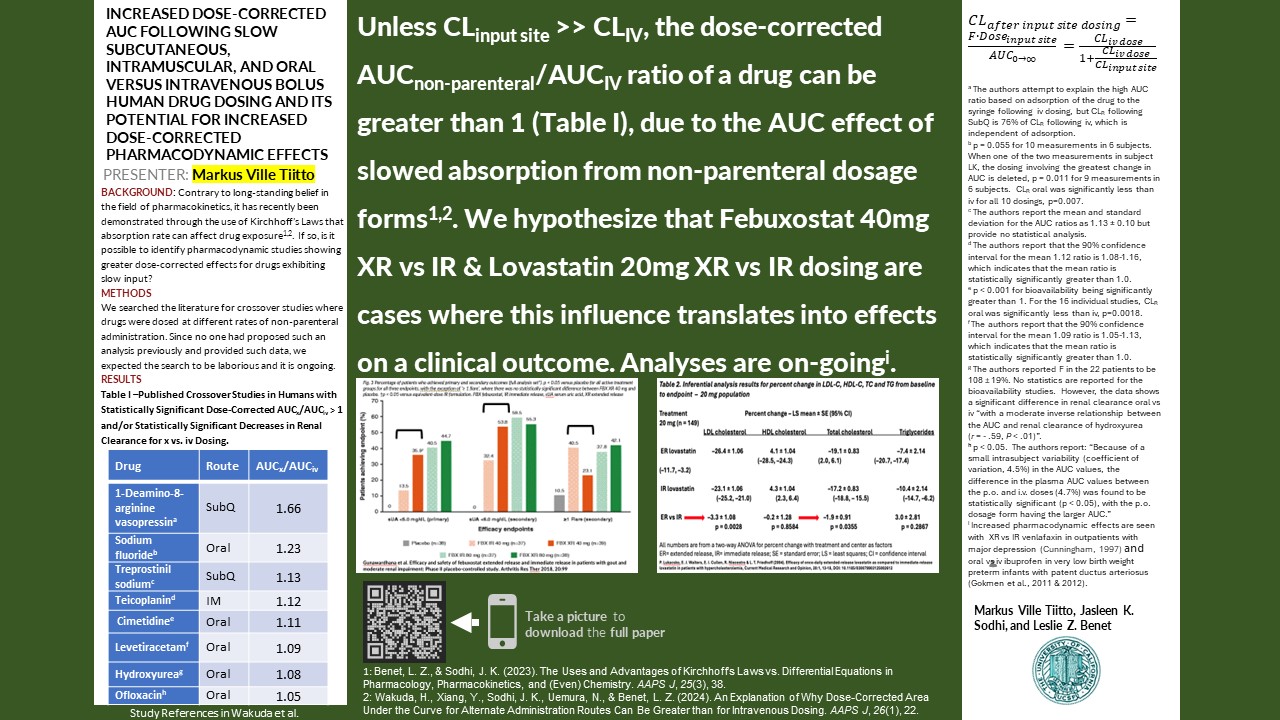
The descriptive text for the Supplementary Material is:  Poster PII-122 presented at the March 26-29, 2024, meeting of the American Society for Pharmacology and Experimental Therapeutics.
